# AIoT-Enabled Rehabilitation Recognition System—Exemplified by Hybrid Lower-Limb Exercises

**DOI:** 10.3390/s21144761

**Published:** 2021-07-12

**Authors:** Yi-Chun Lai, Yao-Chiang Kan, Yu-Chiang Lin, Hsueh-Chun Lin

**Affiliations:** 1 Department of Public Health, China Medical University, Taichung 406040, Taiwan; u106026475@cmu.edu.tw (Y.-C.L.); u106026477@cmu.edu.tw (Y.-C.L.); 2 Department of Electrical Engineering, Yuan Ze University, Chung-Li 32003, Taiwan; yckan@saturn.yzu.edu.tw; 3 Department and Institute of Health Service Administrations, China Medical University, Taichung 406040, Taiwan

**Keywords:** AIoT, machine learning, SVM, ANFIS, HHT, lower-limb rehabilitation exercise

## Abstract

Ubiquitous health management (UHM) is vital in the aging society. The UHM services with artificial intelligence of things (AIoT) can assist home-isolated healthcare in tracking rehabilitation exercises for clinical diagnosis. This study combined a personalized rehabilitation recognition (PRR) system with the AIoT for the UHM of lower-limb rehabilitation exercises. The three-tier infrastructure integrated the recognition pattern bank with the sensor, network, and application layers. The wearable sensor collected and uploaded the rehab data to the network layer for AI-based modeling, including the data preprocessing, featuring, machine learning (ML), and evaluation, to build the recognition pattern. We employed the SVM and ANFIS methods in the ML process to evaluate 63 features in the time and frequency domains for multiclass recognition. The Hilbert-Huang transform (HHT) process was applied to derive the frequency-domain features. As a result, the patterns combining the time- and frequency-domain features, such as relative motion angles in y- and z-axis, and the HHT-based frequency and energy, could achieve successful recognition. Finally, the suggestive patterns stored in the AIoT-PRR system enabled the ML models for intelligent computation. The PRR system can incorporate the proposed modeling with the UHM service to track the rehabilitation program in the future.

## 1. Introduction

Home rehabilitation care serves many people with chronic disease with body degradation, and it is imperative in an aging society [1]. Physicians design physiotherapy programs for patients to take regular rehabilitation exercises to reduce pain and restore physical functions. For example, programs are designed for stroke patients who have lower-limb hemiplegia, which usually causes abnormal gait due to the affected joints, such as the moment of hip landing, the maximum extension angle when standing, the joint angle when the toe is off the ground, the maximum flexion angle of the stepping, the range of knee joint angle, etc. [2]. Improper muscle movement patterns for hemiplegia legs can lead to poor compensatory gait. Correct lower-limb rehabilitation exercises, such as hooking leg and padding toe, can increase the angle range of knee flexion and ankle dorsiflexion of the patient to enhance their walking stability [3,4]. With innovative medical technology, physicians can remotely track the routine home rehabilitation records of the outpatients to reduce clinics and save medical resources [5,6].

The past studies developed the sensing and monitoring tools to facilitate treatment and care of persons with lower limb loss [7]. The conventional rehabilitation systems with multiple sensors of monitoring angulation data distinguished facilitating characterizations of amputee activity for sitting, standing, and walking outside the clinic [8,9]. The novel robotic systems also involved multiple sensors in analyzing explicit and implicit information such as spatial distribution and pulse information for rehabilitation [10]. However, these systems, which require expertise for operation, are inappropriate for patient use in the home. A single inertial sensor performs better at exercise evaluation in the home-healthcare management cases than combining several sensors [11,12].

Network technology can protect patient’s privacy in home health care by using non-imaging and non-invasive equipment to measure health data ubiquitously [13]. The physical therapy program with ubiquitous healthcare management (UHM) enabled clinicians to track the records of patient’s home rehabilitation exercises remotely [14,15]. The UHM system integrates cloud computing with the artificial intelligence of things (AIoT) functionality through data preprocessing, feature extraction, and data training to recognize and track patients’ movements. A monitoring system with artificial intelligence (AI) technology was developed to recognize stable personalized rehabilitation exercises patterns for assessing outpatient self-therapy progress at home [16]. The past studies applied the wearable sensors with embedded accelerometer and gyroscope to measure acceleration and angular velocity, which can derive the spatial components such as relative angle, angular velocity, acceleration, and their rates of change, etc. in the time domain, to enable machine learning (ML) for the posture and movement features before recognition [17,18].

Spectrum analysis is a necessary process to obtain the frequency-domain features for the ML methods. The well-known fast Fourier transform (FFT) can calculate the overall signal spectrum, which defines the main frequency corresponding to power spectral density (PSD) [19,20]. In advance, Hilbert-Huang transformation (HHT) is an algorithm to decomposed unstable and non-linear signals into several sine waves with approximate periods and unfixed amplitudes [21]. For analysis, the HHT drives empirical mode decomposition (EMD) to calculate intrinsic mode functions (IMFs) and yield a Hilbert spectrum (HS), which combines instantaneous frequency and energy in a time-frequency domain [22]. The past studies for electrocardiography (ECG) analysis employed the HHT to produce the HS corresponding to the IMFs, then integrated the frequencies along the time axes to obtain a marginal Hilbert spectrum (MHS or HMS) that provides the possible features similar to the PSD [23,24,25].

The AI-enabled measurement modeling is used to challenge the terms of sensor selection, result performance, and recognition efficiency. The design requested techniques to process samples featuring, machine learning, sensor measuring, stream data classification, and a trackable system [26]. The conventional AI-compliant ML model conducted data classification algorithms with either dimension separation or neural networks, such as support vector machine (SVM) and adaptive neural fuzzy inference system (ANFIS). The SVM, consisting of kernel function and hyperplane, is a typical classifier based on binary classification [27,28]. The kernel function projects the two sets of feature vectors into high-dimensional space and yields the hyperplane to divide the datasets. Both sides of the hyperplane can support the closest points for two datasets with the maximum boundary from the hyperplane. The ANFIS, including a Fuzzy set and the logic rules based on the Fuzzy theory, is a neural network (NN) with a Sugeno-type FIS for estimation [29]. The Fuzzy set consists of the membership functions (MFs) of geometric shape to formulate the degree of the involved elements corresponding to the features, and the logical rules control the input MFs relating to the output targets [30,31].

Our previous study established a prototype of personalized rehabilitation recognition (PRR) system with ANFIS modeling to recognize the decomposed motions of the scheduled upper-limb rehabilitation exercises and reached the average identification rate of over 90% [32]. The modeling drove an initial fuzzy inference system (FIS) with fuzzy logic rules to train the sample datasets of the scheduled motions via the NN-based process. The self-developed recognition module was then installed in the mobile APPs for AIoT application. This study will extend the previous work to improve the PRR system, which can integrate AIoT functionality with the SVM and ANFIS models, to track the complete exercises of hybrid lower-limb rehabilitation for the UHM services.

## 2. Methods

**Figure 1:**
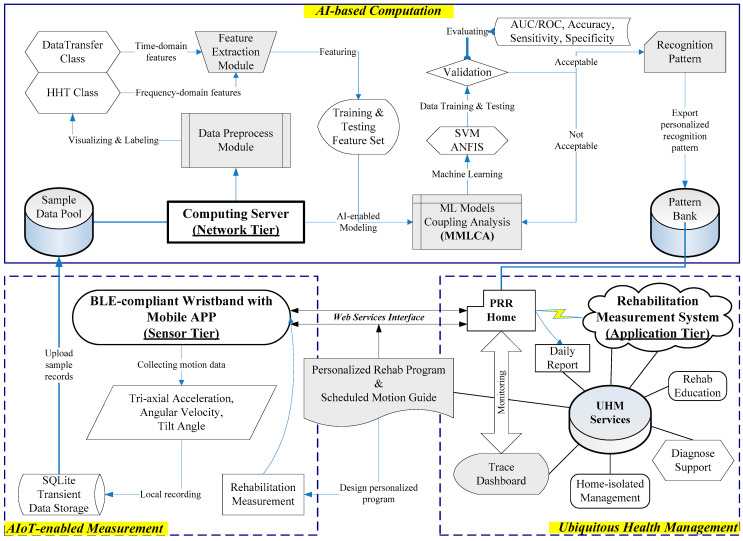
The three-tier infrastructure of the AIoT-enabled personalized rehabilitation recognition system.

The proposed AIoT-enabled PRR system comprises the three-tier infrastructure as shown in Figure 1. They are (1) a sensor tier for AIoT-enabled measurement, (2) a network tier for AI-based computation, and (3) an application tier for ubiquitous health management.

The MetaWearC sensor, the previous model of MetaMotionC reference to the website (https://mbientlab.com/metamotionc/, accessed on 10 July 2021), was manufactured by Mbientlab Inc. The specification is available for download from the website. (https://mbientlab.com/documents/MetaWearC-CPRO-PS.pdf, accessed on 10 July 2021) The MetaWearC includes the BMI160 chip, which embeds the 6-axis accelerometer and gyroscope. The accelerometer and the gyroscope can detect the moving object’s acceleration and angular velocity in the x, y, and z axes, respectively. Therefore, the tilt angle can be derived from the acceleration, as shown in Equation (1) for measurement.

(1) $$\left(\begin{array}{ccc} \tan\theta_{x} & \tan\theta_{y} & \tan\theta_{z} \end{array}\right)=\left(\begin{array}{ccc} \frac{a_{x}}{\sqrt{a_{y}^{2}+a_{z}^{2}}} & \frac{a_{y}}{\sqrt{a_{z}^{2}+a_{x}^{2}}} & \frac{a_{z}}{\sqrt{a_{x}^{2}+a_{y}^{2}}} \end{array}\right)$$

(2) $$\left(\begin{array}{ccc} \sin\theta_{x} & \sin\theta_{y} & \sin\theta_{z} \end{array}\right)=\frac{1}{\sqrt{a_{x}^{2}+a_{y}^{2}+a_{z}^{2}}}\left(\begin{array}{ccc} a_{x} & a_{y} & a_{z} \end{array}\right)$$

where measured acceleration be a spatial vector a, and the component (*a_x_*, *a_y_*, *a_z_*) is with reference to gravity g (i.e., the acceleration unit is g). We calculated two types of tilt angles *θ* in Equation (1) for reference. Equation (1) shows the inclinometer’s formulation, which calculates the projected angle components regarding the vector corresponding to the plane. Equation (2) presents the included angles between the vector and the base axes used to compute the angle by two accelerometers in our previous study [13,14]. When we considered the relative angles with respect to the initial position, the results due to both formulas would reach the same distribution for machine learning.

In practice, the sensor can deliver the three-axis acceleration and angular velocity signals to the smartphone or tablet through Bluetooth low energy (BLE) in the sensor tier. The self-developed APP in the mobile device can configure measurement frequency (e.g., 100 Hz) and calculate the tilt angle due to acceleration, then transfer all data to the network and application tiers, respectively, for data training and motion tracking. The web-based system was constructed by Java technology with MATLAB™ toolbox (license no. 40697750) in a computing server with intelligent computation that extracts the features, processes data training, and evaluates the ML models to export the recognition patterns. The personalized patterns can be stored in a pattern bank for application and management.

### 2.1. Lower-Limb Rehabilitation Exercise

**Figure 2:**
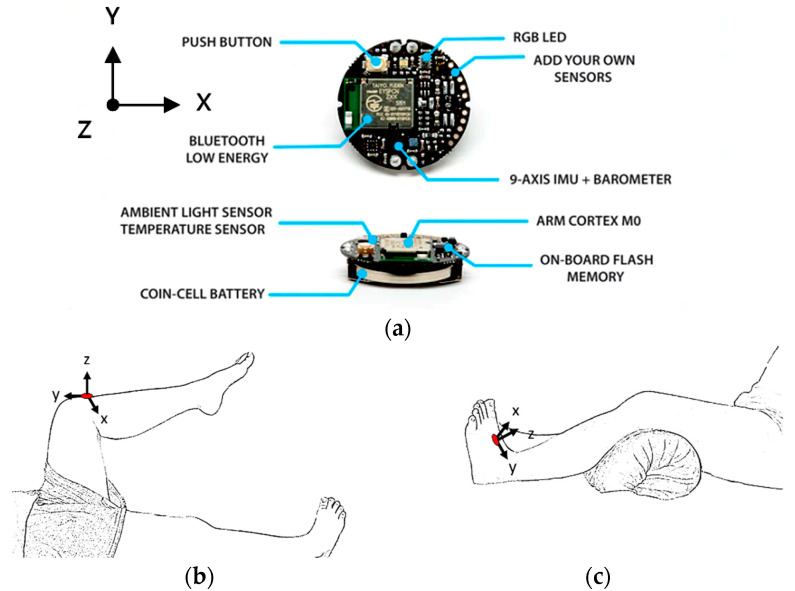
The MetaWearC sensor and positions (**a**) local coordinate for the device (https://mbientlab.com/metamotionc/, accessed on 10 July 2021), (**b**) position at the knee (Ex. 1–4) and (**c**) position at the foot (Ex. 5–6).

We selected six rehab exercises including the schedule of decomposed motions, as shown in Figure 2 and Table 1, for training muscle strength of the lower limbs including (1) iliopsoas muscle, (2) gluteus medius, (3) quadriceps femoris, (4) hamstrings, (5) tibialis anterior, and (6) triceps surae exercises (Ex.). The subject can carry out exercises on the bed or chair for measuring the postures of lower limbs by wearing the sensor at the specified position [33,34].

In the study, the authors, Lin and Lai, as the subjects A and B, respectively, wore the device at the sensor tier to collect rehab sample datasets for the AIoT measurement. As shown in Figure 2, the sensor was attached to the knee for Ex. 1-4 and on the instep for Ex. 5 and 6. We experienced that the sensors on both positions can deliver stable and regular signals for analysis. The tester must perform several cycles of each exercise according to the guidelines defined in Table 1 and Table 2. Each exercise cycle was completed in 11 s to create 1100 data points according to the design.

### 2.2. AI-Based Recognition Process

**Figure 3:**
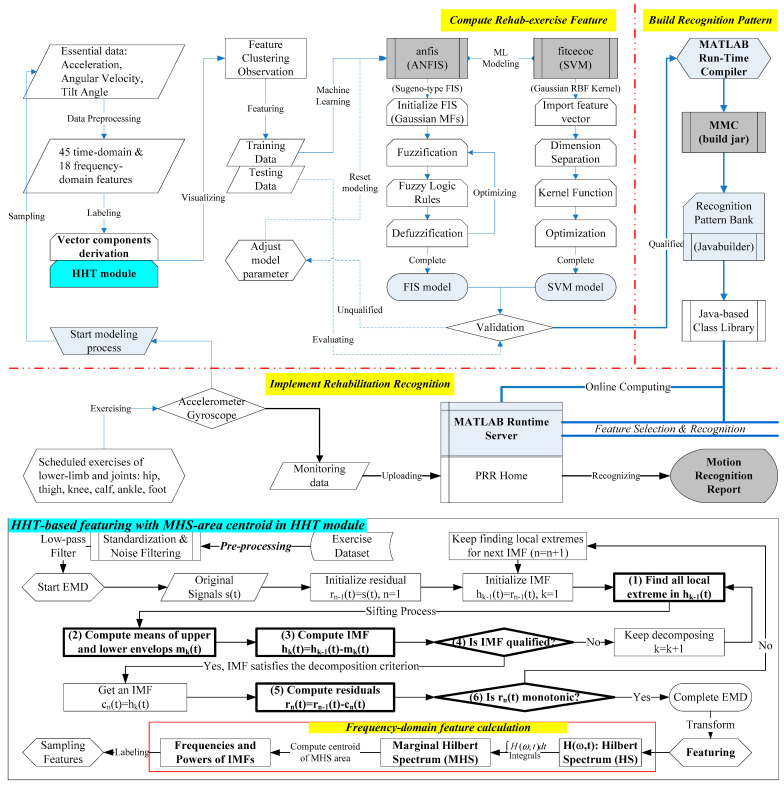
The AI-enabled machine learning and the HHT-based featuring flowchart.

The computing server at the network tier drives AI-based computation, including data preprocessing, feature extracting, machine learning, and modeling, as shown in Figure 1, to generate the recognition patterns for the application tier. To enhance the computing progression, we bundled an analytical module “MMLCA” to drive the multiple ML models (e.g., SVM and ANFIS) for coupling analysis of the features. Finally, the recognition model of the personal motion pattern can be compiled to a Java-based class through MATLAB^TM^ compiler and be compressed in a class library (i.e., the jar file) for the pattern bank of runtime server in the PRR system. The main processes in the PRR modeling, as shown in Figure 3, include (A) compute rehab-exercise feature, (B) build recognition pattern, and (C) implement rehabilitation recognition.

#### 2.2.1. Compute Rehab-Exercise Feature

(1) *Data preprocessing and sample labeling*. We used relative data, which subtracts the values measured at the initial position from that at the motion position, instead of raw measurement in the exercise for data preprocessing to eliminate the errors due to the difference between individual behaviors. Similarly, the relative angle, relative acceleration, and relative angular velocity were defined by the tilt angle, acceleration, and angular velocity between the measured motion point and the initial point. With the design of cyclical exercise, an 11-s segment contained the relative data of an exercise cycle and was labeled by the exercise ID. We observed that the relative angle presented a significant difference comparing with other components measured for the stable rehab motions. Therefore, we adopted the angle-related features in this study. Then, the segment data can be transformed into various features as shown in Table 3.

(2) *Featuring*. In the study, we derived 15 time-domain and 6 frequency-domain features, as shown in Table 3, from three-axis components of the relative angle data of the rehab motions for machine learning, i.e., a total of 63 features (=21 × 3) were available in the computation. The feature symbol with xi, yi, zi means the i-th feature corresponding to the x-, y-, z-axis component, respectively. For example, the feature (1) xsum, as shown in Table 3, represents the summation of all x-axis angle data points in a segment. We created the “DataTransfer” module to transfer the time-domain features suggested by the past study [40] from the motion data. In addition, the “HHT” module, as shown in Figure 3, processes the EMD function to compute the IMFs of each segment and transform the correspondent HS via the HHT function, which the essential steps are detailed below.

The HHT process includes the following six steps in each decomposition cycle. They are: (1) Find the upper and lower envelopes using a cubic spline that sketches the local maximum and minimum to each peak and trough in the waveform, respectively. (2) Compute the average of both envelopes to get the mean envelope. (3) Subtract the difference between the original signal and the mean envelope to get the component function. (4) Check whether the component function satisfies the sifting criterion or not, e.g., the number of extreme values is the same as the number of zero crossings; if not, then use this component function as the original signal and repeat (1) for the next screening until it meets the criterion, and then get an IMF component. (5) Subtract the original signal from the IMF component to obtain the residue function. (6) If the residue function is monotonic, then the decomposition is completed. If not, it can still be decomposed, and so the residue function should be taken as the new signal data, and steps (1) to (5) should be repeated to obtain the next component.

Then, the module integrates the HS along the time axis to obtain the marginal Hilbert spectrum (MHS), and the centroid of the MHS area reveals the frequency and energy for the frequency-domain features. In which, we took the first three IMFs for the study since the latter-order IMFs were similar. The symbol IMFj_f and IMFj_e (*j* = 1, 2, 3) represent the frequency and energy, respectively, regarding the MHS-area center of the *j*-th IMF. For example, the frequency of the first IMF in the x-axis is xIMF1_f. We compared the total 63 features of both domains in coupling analysis for different exercises to find the proper features. In addition, if each segment’s duration is different, the power (i.e., energy divided by duration) can be considered for consistency.

(3) *ML modeling*. I. SVM model. The SVM essentially follows three steps in terms of analysis as shown in Figure 3; they are (1) dimension separation of the feature space, (2) inner product of the kernel function, and (3) optimization of data separation. The method formulates the objective function to optimize the boundary (i.e., the support vector) with error minimization between different dimensions in a feature space. Past studies have suggested the categorical features for good efficacy [41]. Kernel function for nonlinear projection is required to reduce multivariate dimensions of the features. In theory, the kernel function usually defines two eigenvectors based on the linear, polynomial, or Gauss radial basis function (RBF) to identify the similarity of the features [42]. In this study, the SVM models employed the MATLAB^TM^ module “fitcecoc” with the “templatesvm” learner and the three-order polynomial kernel function to classify six rehabilitation exercises. The important parameters including “kernel scale parameter (the coefficient to define how far the influence of a single training example reaches),” “box constraint (the regulation parameter to control the tradeoff between margin maximization and error minimization)”, “coding design mode (to reduce the multiple labels to a series of binary classes)” were denoted in Table 4. In addition, we substituted a variety of feature patterns (i.e., including partial or all features) to train the models for evaluating the proper features.

II. ANFIS model. The ANFIS takes the crisps as output’s MFs for the Sugeno-type FIS to process NN-based iteration, as shown in Figure 3, with root mean square error (RMSE) until optimization [43]. The initial FIS needs *n^m^* Fuzzy logic rules with the conventional grid partitioning method that assigns *n* MFs for each input of the *m* features in the Fuzzy set. However, too many rules can slow down the computation speed and even run out of memory. Besides, the FIS is unable to infer the output if the input values exceed the range of the MFs. We then employed the modern subtractive clustering method to reduce the rules, which estimates the number of clusters in the input dataset based on specified influence range and squash factor to assign a Gaussian-type MF in a cluster [44]. The calculation explores each clustering’s density in the feature dimensions and takes the largest cluster’s index as the cluster center. The center is subtractive from the data point to recalculate the density index for a new cluster center. The clustering generates the same amount of clusters for each input, and the same-order MFs in each input formulate one rule. For example, we setup the influence range of 0.8, and the squash factor of 1.25 for clustering the 18 frequency-domain features (i.e., iIMFj_f and iIMFj_e, *i* = x, y, z, *j* = 1–3) and each input dataset can be subtractive into 9 clusters.

We employed the MATLAB^TM^ functions “fitcecoc” and “anfis” to generate the SVM and ANFIS models, respectively, and then used “predict” and “evalfis” to recognize the data for the models. The model’s parameters are suggestive in Table 4 for machine learning. For enabling the binary-based SVM with multiclass recognition capability, the “fitcecoc” module provides several coding design modes such as one-versus-one (OVO), one-versus-all (OVA), ordinal, binary/ternary complete, and dense/sparse random, to reduce the multiclass problem to a series of binary problems. We compared these modes and explored the best option with the OVO mode, which takes a binary pair for positive and negative classes but ignores the rest. Then, the SVM model calculated the support vectors to obtain posterior probability corresponding to the target labels in classification. Only one label with a probability approaching 1.0 is the predicted class. For example, if the six exercises’ posterior probabilities were {0.02, 0.01, 0.03, 0.05, 0.04, 0.98}, then the exercise with label #6 was recognized. On the other hand, the ANFIS model inferred an approximate value around the target label. The value after rounding can be the predicted class. For instance, if the estimated output is located between 0.5 and 1.4, label #1 exercise was recognized.

(4) *Validation.* The subject obeyed the motion guide to take six types of rehabilitation exercises. The testing data validated the model with data training to either export the optimal model or adjust the parameters for remodeling. We applied the confusion matrix (CM) and the receiver operating characteristic (ROC) curve to evaluate the SVM and ANFIS models for multiclass recognition. Equation (3) formulates the evaluation equations related to the CM and ROC. The CM, which including the number of true-positive (TP), true-negative (TN), false-positive (FP), and false-negative (FN), implies the accuracy, sensitivity, and specificity to reveal the model’s capability for recognition. The sensitivity and specificity construct the basis of the ROC curve, in which the area under the curve (AUC) is a criterion to calculate the effectiveness of predicting positive and negative labels [45]. We used the ROC curves based on the one-versus-rest (OVR) type to evaluate the model’s performance for multiclass recognition; i.e., the curve exhausts all combinations of positive class assignments by taking one target label (as positive) with others (as negative). In this study, we took one exercise as the positive label versus the rest as the negative label to perform six ROC curves of the rehab exercises.

ACC (accuracy) = (TP + TN)/(TP + FN + FP + TN)

TPR (true-positive rate) = TP/(TP + FN) = Sensitivity(3)

FPR (false-positive rate) = FP/(FP + TN) = 1 − Specificity

#### 2.2.2. Build Recognition Pattern

The process, as shown in the right block in Figure 3, employed a MATLAB^TM^ compiler (named MMC Apps) that can convert the recognition model to a Java class library (named “jar” file) for the MATLAB^TM^ runtime server (MRS). The jar files are stored in the pattern library (named “jarlib”) of the PRR system. We designed three types of jars, including “FeatureTransform (FT)”, “PatternRecognition (PR)” and “DataConvert (DC)” classes in the jarlib to incorporate the models with the “javabuilder”, which is the jar driving the patterns in the MRS for intelligent computation.

#### 2.2.3. Implement Rehabilitation Recognition

Two routes of dataflow, including modeling the pattern and tracking the motion, are available for implementing rehabilitation recognition. The sensor firstly sends the lower-limb exercises’ signals based on the motion guide to start the modeling process for computing rehab-exercise features and building recognition patterns. Once the specific patterns are stored in the recognition pattern bank, the sensor can deliver the measured data to the PRR system incorporating the MRS with the correspondent pattern for recognition. Finally, the system exhibits the traceable diagram on the dashboard interface to manage the motion tracking for rehabilitation.

### 2.3. Rehabilitation Recognition System

The lower-right block of the architecture, as shown in Figure 1 displays a prototype of the rehabilitation measurement system for the UHM services. This part expanded our previous study by integrating the Java-based PRR system with the MRS to implement real-time computation with AI-enabled modules. In the PRR system, we designed the repository including data_log, patt_bank, out_log, and fig_log to save input features, recognition patterns, output labels, and traceable diagrams, respectively. The dataset name contains unique symbols, such as identification number, recording timestamp, and pattern index, for managing the personal tracing data. When recognizing the exercise in the infrastructure as shown in Figure 1, the sensor tier collects and delivers the motion data to the data_log of the PRR system. The FT class can transform the data as the time- and frequency-domain features. The PR class then activate the specific model corresponding to the selected feature set for the recognition process. Finally, the DC class transforms the output labels of the targets to the Java-complied data format for the traceable diagram on the Web dashboard. In addition, the dashboard can provide the rehabilitation diary and healthcare education for references of the clinical diagnoses in the UHM services.

## 3. Results

**Figure 4:**
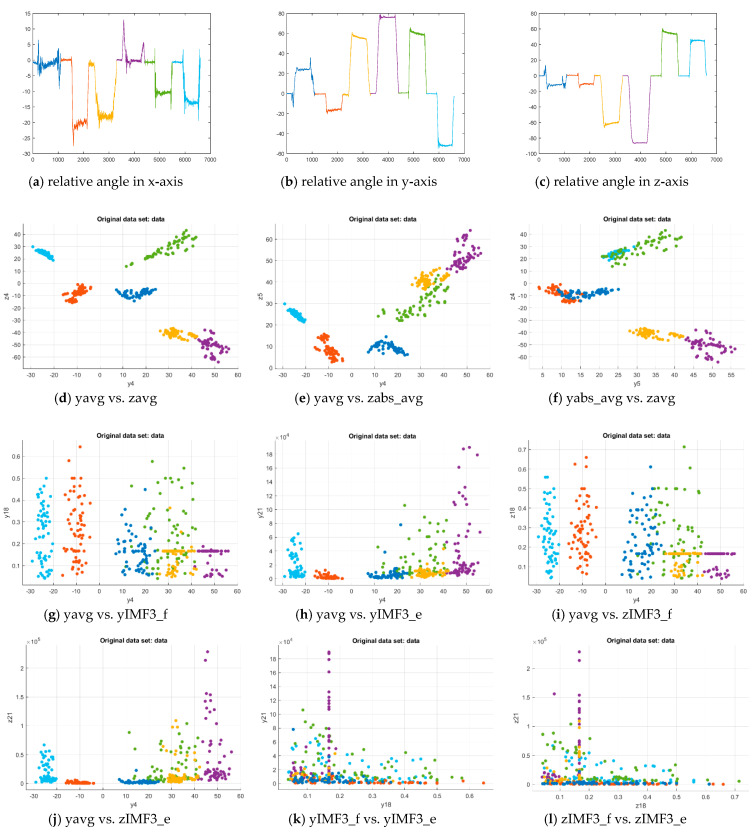
Clustering comparison of the specific feature pairs corresponding to six exercises. Label color: Ex. 1 (blue), Ex. 2 (red), Ex. 3 (yellow), Ex. 4 (purple), Ex. 5 (green), Ex. 6 (cyan).

We designed the modeling process with a laboratory scale test because of a limited budget and devices. The modeling refers to the typical physical therapy that requests the patient to do personalized rehabilitation exercises based on the motion guide. To enhance the modeling effect in machine learning, we employed the k-fold cross-validation method to evaluate the model with a few samples to complete data training and validation for approving the model’s parameters for various feature patterns before the testing process. In practice, Subject A obeyed the motion guide of each rehab exercise to generate 95 segments that can be further split into 65 segments for 5-fold cross-validation and 30 segments for testing. A time segment contained 1100 data points with a sensor frequency of 100 Hz for the exercise scheduled in an 11-s cycle. Each segment can be transferred to a dataset with all features. Then, the six exercises due to Subject A included 390 and 180 datasets for cross-validation and testing, respectively, in the design. The pattern’s model can be obtained by training the same datasets again with approved parameters for further testing. Additionally, Subject B also produced 30 segments of each exercise based on the same motion guide to verify the model’s recognition capability on another subject’s motions. By observing data clustering distribution as shown in Figure 4, we adopted the potential feature sets according to the variables shown in Table 3 for machine learning.

The models’ accuracies due to 5-fold cross-validation were shown in Table 5. Then, the model was retrained by the 390 datasets with fine-tune parameters, and tested by the 180 datasets for further evaluation. Subject B provided another 180 datasets for testing to verify the feasibility of the different subjects. The results were presented in Table A1 in Appendix A. As shown in Table 5, the patterns present the SVM and ANFIS models with the proper feature sets for the exercises. In addition to all 3-axis features in both domains, we explored the qualified feature sets based on y- and z-axis variables, such as sum, the sum of absolute, average, and average of absolute in the time domain, and that plus frequency and energy of IMFj (*j* = 1−3) due to MHS area in the frequency domain. With 5-fold cross-validation, the patterns (1), (3), (5), (6), (8), and (12) could achieve an accuracy as high as 0.99. These patterns also attained the OVR-type AUC of 1, as shown in Table A1, for Subj. A’s testing data (i.e., the personalized training data). In which the patterns (3) and (6), as well as the patterns (5) and (8), contain similar properties (i.e., sum and average of data group). We then validated the model’s capability by applying the patterns (1) and (12) for SVM, as well as the patterns (3) and (8) for ANFIS, to recognize Subj. B whose testing data could include the different range of motion (ROM) from the trained data.

### 3.1. SVM Model

**Figure 5:**
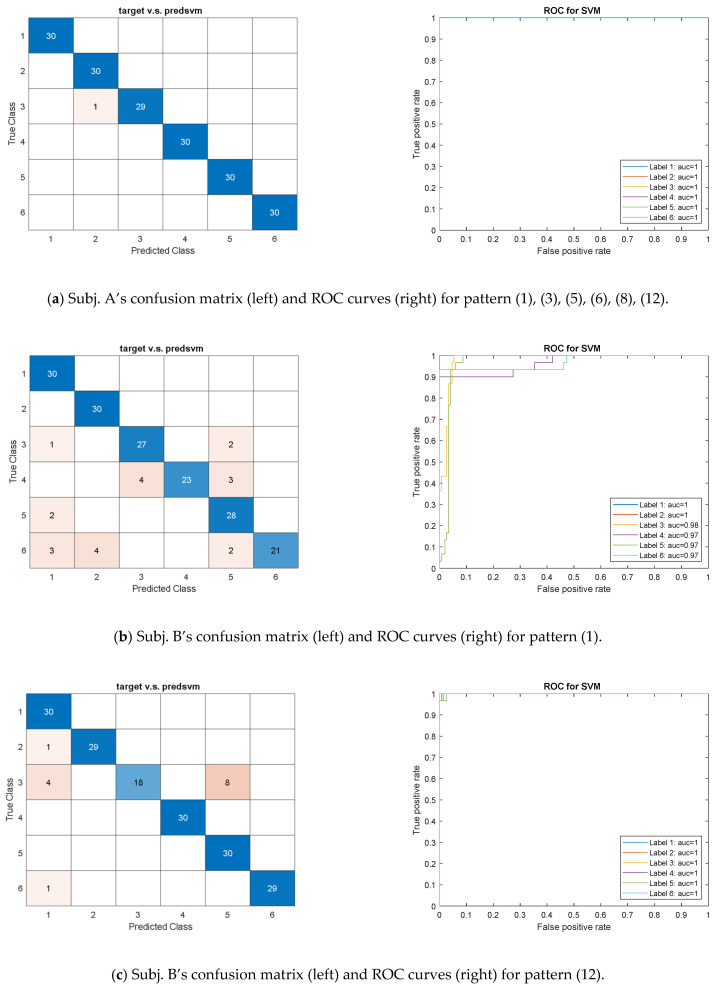
The confusion matrix and one-vs-rest ROC curves for validation dataset using the SVM model with (**a**) feature pattern (1), (3), (5), (6), (8), and (12) for Subject A, (**b**) feature pattern (1), and (**c**) feature pattern (12) for Subject B.

The feature pattern (1), (3), (5), (6), (8), and (12) trained by the SVM model displayed the almost perfect CM and ROC curves, as shown in Figure 5a, for recognizing Subject A’s six exercises. As shown in Figure 5b, the pattern (1) that provided 63 features for Subject B’s six exercises, could achieve AUCs value higher than 0.97. The design of rehabilitation exercises consists of stable and slow motions, which produce a similar MHS-area center via HHT and lead recognition worse than that by the time-domain features. Meanwhile, the features related to the y and z axes were more sensitive than the x-axis variables because the exercises were designed on the same plane. If the motion variation along the x-axis must be identified in practice, then the feature set should include the x-axis features. As shown in Table A1, the IMF3 contributed more effect features than IMF1 and IMF2 by comparing the AUCs for the pattern (9), (10), and (11), which include the features of decomposed modes due to the HHT.

In comparison with Subject A, we evaluated the pattern (3), (5), (6) and (8) that provide time-domain features for Subject B with lower AUCs as shown in Table A1. We then employed pattern (12), which combined pattern (8) with the frequency-domain features (i.e., IMF3-related frequencies and energies) to improve the model’s performance. As shown in Figure 5c, the results illustrate the excellent CM and OVR-type ROCs with the AUC approaching 1. The successful recognition approved the pattern’s efficacy for the subjects, and allowed to play the scheduled exercises with slightly various ROM.

### 3.2. ANFIS Model

The ANFIS model was unsuitable for training many features because the number of Fuzzy logic rules was restricted to computation memory. Therefore, pattern (1), which needs over 250 rules for building the FIS with subtractive clustering was not applied for this model. In addition, the model did not pass cross-validation for pattern (15) because many test data exceeded the MF’s inferring range as training in each fold.

**Figure 6:**
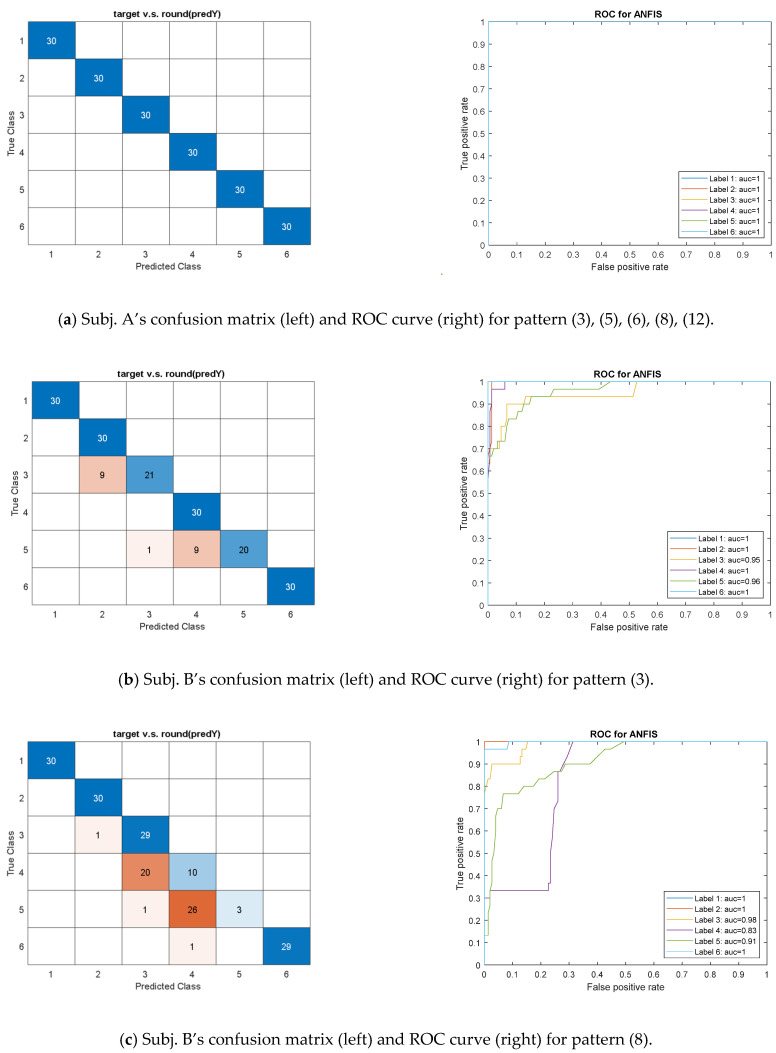
The confusion matrix and one-vs-rest ROC curves for test and validation dataset using the ANFIS model with (**a**) feature pattern (3), (5), (6), (8), (12) for Subject A, (**b**) feature pattern (3), and (**c**) feature pattern (8) for Subject B.

We then considered other patterns that include few features to initialize the FIS for training. In calculation, the ANFIS model inferred the output within a range of labels corresponding to the exercise ID (e.g., the output value of 1.75 stands for the estimated exercise ID between labels #1 and #2). As shown in Figure 6a, patterns (3), (5), (6), (8), and (12) were available for Subject A to perform the perfect ROCs with the AUC of 1 and the CM with the diagonal elements only. When validating the patterns with Subject B, pattern (3) successfully recognized four exercises except for Ex.3 and 5 due to the CM as shown in Figure 6b, while the OVR-type AUCs were over 0.96.

Additionally, the CM, as shown in Figure 6c, reveals that the ANFIS with pattern (8) mostly recognized Subject B’s Ex. 1, 2, 3, and 6 but badly misjudged the Ex. 4 and 5. Subject B played the motions related to knee and foot, respectively, in the Ex. 4 and 5, most likely out of the ROMs corresponding to Subject A’s training data. The different subjects usually played their personal ROMs for these two exercises. Compared with other patterns, pattern (8) was not entirely accurate for the ANFIS to identify Subject B’s Ex. 4 and 5, but their OVR-type AUCs still reached reasonable rates at 0.83 and 0.91, respectively. Regarding the ANFIS with pattern (15), which was failed in cross-validation, the trained model still recognized Ex. 1, 2, and 3 with the AUCs over 0.9, as shown in Table A1. The suggestive patterns can approve the feasibility and reliability of the models for the designed rehab exercises.

### 3.3. Validation and Application

**Figure 7:**
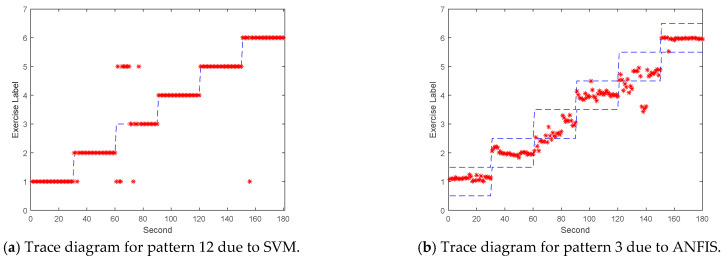
The examples for subject B’s traceable diagrams due to the SVM and ANFIS models with the diverse feature patterns: (**a**) SVM with pattern (12), (**b**) ANFIS with pattern (3).

The dataflow as shown in Figure 2 finally illustrates the recognized exercises on the traceable diagrams as shown in Figure 7. The dash lines in the diagram represent the label bounds of the scheduled motions. The SVM model predicts the exercise with the integer label and the correct label is pointed on the line. The ANFIS model estimates the output by a numeric crisp value and the correct crisp will locate within the range of the label bounds. As shown in Figure 7, both models illustrated the realistic tracking diagrams for the patterns (3) and (12) as validating the 180 segments due to subject B’s testing data. The validation approved the model’s accuracy for tracking the various people who obeyed a scheduled motion guide to practice the exercises for application. If the subject completely follows the guide, the time-domain features can successfully recognize the designed rehabilitation exercises. The modeling requested the appropriate feature set for the subject’s motions not fully compliant with the designed schedule. For example, the Ex. 5 is not easy for the subject to keep the foot stably within the ROM of the requested gait to yield the outliers. Therefore, the modeling suggested allowable tolerance for diverse training data and used the pattern with hybrid features in time and frequency domains to improve recognition efficacy.

**Figure 8:**
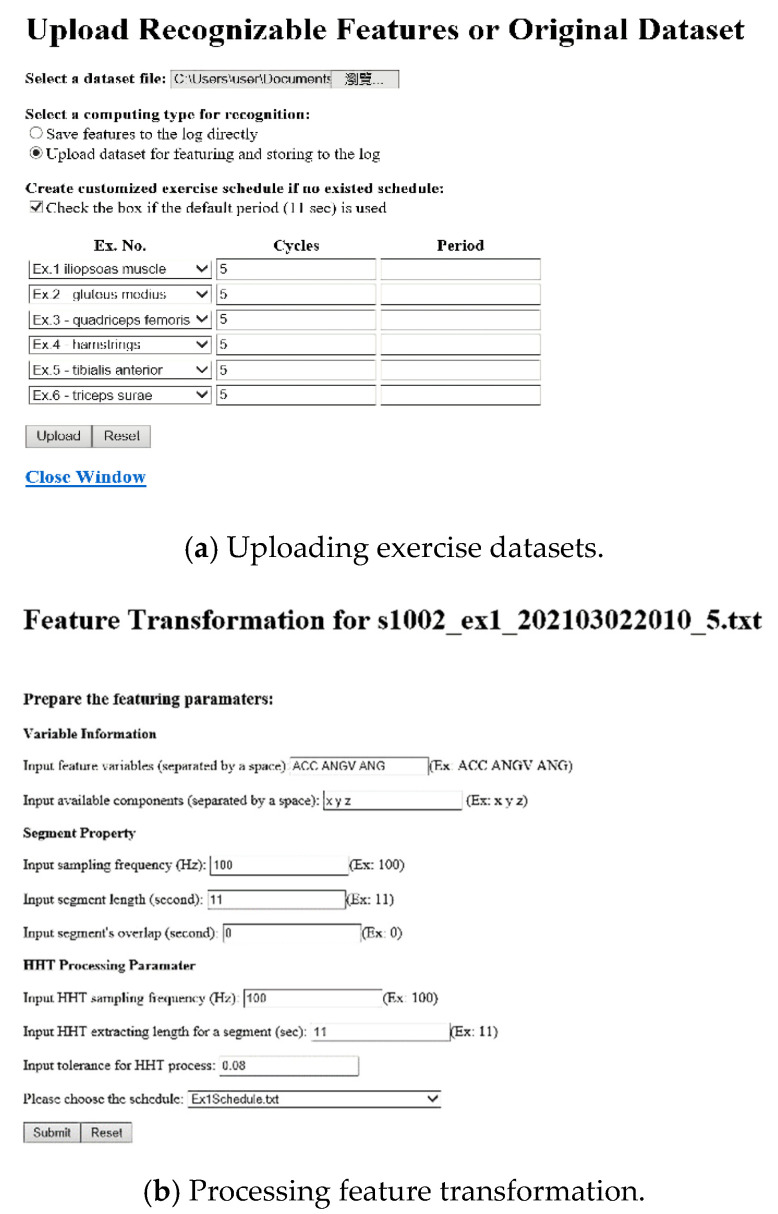

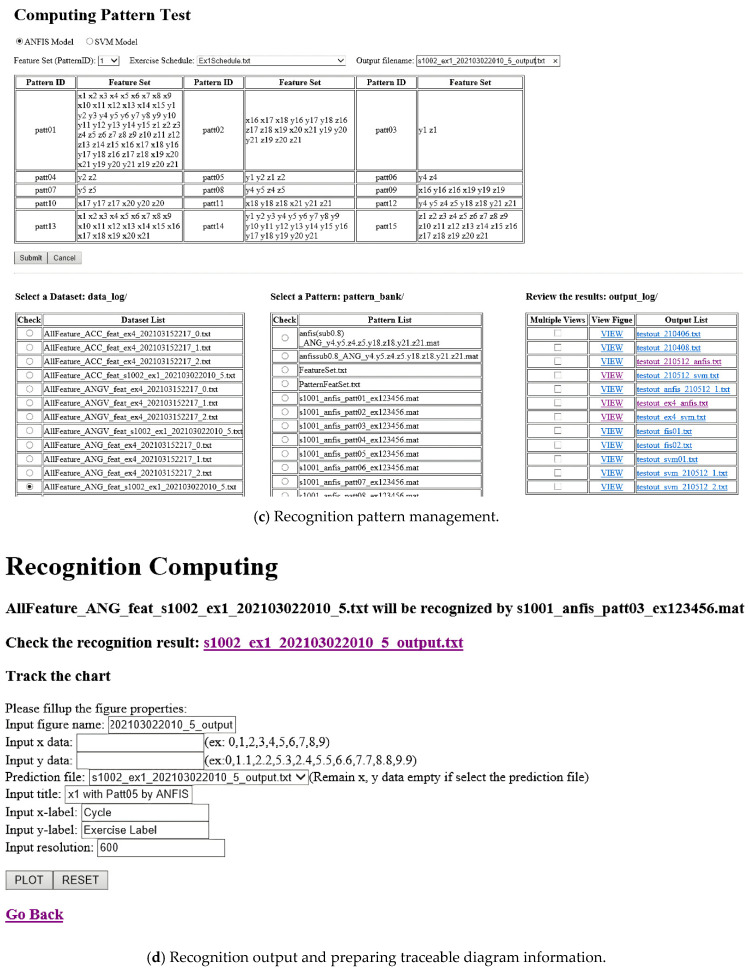

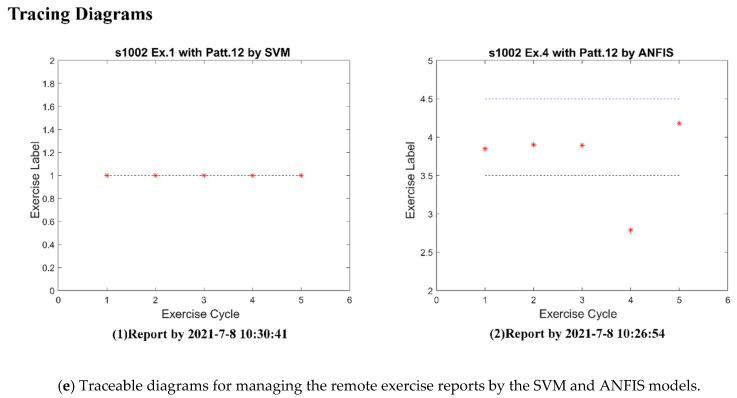
Core interface snapshots of the AIoT-enabled PRR system prototype for rehabilitation recognition management.

We finally implemented the recognition patterns to the self-developed PRR system prototype for the application. The subject uploads the measurement data to the system and assigns the dataset to the specific data log. The manager can transform the data to the proper feature set and select the corresponding model patterns for recognition. The user can easily explore the traceable diagrams for the UHM services through user-friendly interfaces, as shown in Figure 8. The cloud site can acquire the measurement data, either the transformed features or the raw dataset, with the associative exercise schedule via the upload interface as shown in Figure 8a. Figure 8b shows that the feature transformation interface requests the preprocessing parameters and transforms the segment data into the 63 features in time and frequency domains. Figure 8c shows that the pattern management interface provides the available the ML models and feature patterns (i.e., in the “Select a Pattern” division) for cooperation with the exercise schedule and the potential features (i.e., in the “Select a Dataset” division). The interface also presents the list of traceable diagrams (i.e., in the “Review the results” division) for management including the single and multiple views as well as the output data. The pairs of the selected exercise, schedule, patterns and ML models are transferred to the cloud that drives the modules with the MRS for recognition computation. The computing results are returned to the feedback interface as shown in Figure 8d to input the diagram information. Finally, the user can track the diagrams as shown in Figure 8e for management.

## 4. Discussion

We supposed the patient who needs the personalized therapy should be restricted to regular standard exercise by referring to the past studies related to rehabilitation progress with the wearable sensors [7,8,9]. The self-rehabilitation program, therefore, requests the personalized design in the clinic before implementation at home. In the program’s initial phase, the physician can examine the patient’s condition while the patient practices the exercise in the clinic to determine the sensor’s orientation and position and acquire the training data for modeling.

### 4.1. Finding and Benefit

**Figure A1:**
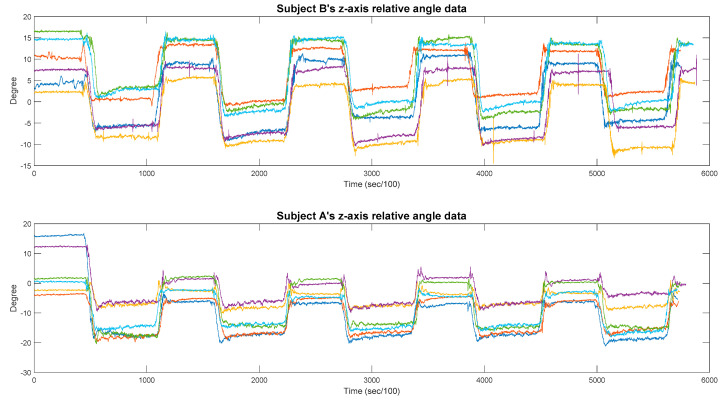
Variation of Ex. 2′s original motion angles in z-axis for subjects A and B. The averages of standard deviation (feature z11) for subjects A and B are 50 and 0.9, respectively, and the absolute standard deviation (feature z12) is 69 and 0.9. The z11 and z12 of training data are 0.9 and 0.9, respectively. Subject A adjusted the motion postures, later on, to resubmit the correct measurement data for improving the analysis.

**Figure A2:**
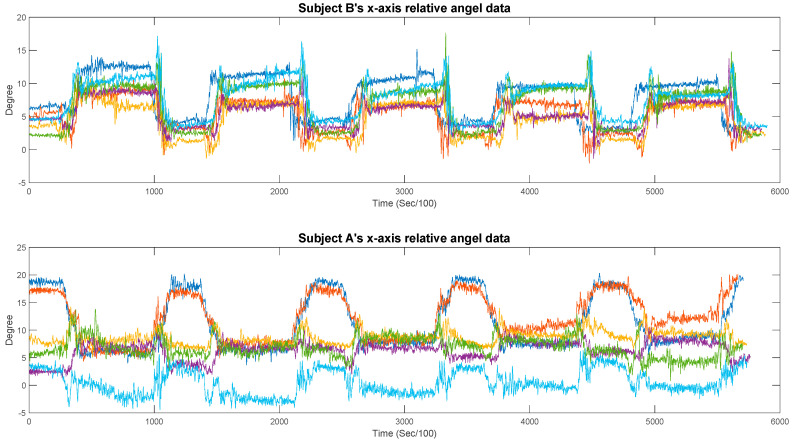
Variation of Ex. 3′s original motion angles in x-axis for subjects A and B. Subject A’s postures were unstable in comparison with Subject B when playing the motions. Subject A adjusted the motion postures later, and then resubmitted the correct measurement data for improving the analysis.

(1) *The pattern with hybrid features in the time and frequency domains enable multiclass exercise recognition*. The past study exhibited good efficacy in recognizing the rehabilitation exercises with the time-domain features [40]. The accuracy due to the CM could be inferior when the subject practiced the exercise slightly different from the motion guide. The pattern with both-domain features overcame the bias to achieve successful recognition, as shown in Figure 5 and Figure 6. The approach enhanced our previous study that only applied the time-domain features to identify the decomposed motions in the FIS model [32]. The current model design explored the patterns with the AI-enabled training process for efficient clinical application.
(2) *Both models can achieve successful recognition with various computing efficacy*. The ANFIS model required computational resources for NN-based iteration, while the input feature’s value must not exceed the bounds of each MF [46]. The model was not suitable for excessive input features to formulate enough Fuzzy logic rules. However, the NN-based iteration allows transfer learning, which reactivates the training process using the existing model by updating new subject’s data for advanced computation to upgrade the capability without rebuilding the model [47]. The SVM model performed efficient computation, but many samples were necessary for classification in the high dimensional feature space. The past study practiced more than 80 features for the 23,000 segment samples to achieve around the accuracy of 0.85 [40]. Therefore, adopting the proper feature sets in the featuring process was important to succeed in the modeling.
(3) *The traceable diagram can help to screen the incorrect motions according to the outlier data.* The diagram displayed the exercise label out of the expected range if the subject did not follow the motion guide or wore the sensor in the wrong position. As shown in Table A1 in Appendix A, the SVM model with the pattern (1) approached the perfect AUCs for both subjects except Ex. 2 and 3 by subject A. As comparing the raw measurement for testing data, as shown in Figure A1 for both subjects, Subject B’s data displayed different variations from subject A’s data after about the 5th second. Reviewing the unrecognizable features (e.g., z11 and z12 related to the angles in the z-axis), Subject A’s testing data revealed the outliers with respect to the training data. We then judged that Subject A most likely adjusted the posture and did not fulfill Ex. 2. Similarly, Subject A played some motion cycles of Ex. 3 with unstable postures, as shown in Figure A2, to affect the recognition performance. When the outliers were removed from the testing data of Subject A’s Ex. 2 and 3, the rest data could be completely recognized.
(4) *The feature patterns imply the model’s computational complexity in relation to recognition.* For the suggested feature sets, the ANFIS model spent 4.3, 7.7, 4.3, 7.8, and 19.3 s, respectively, for the patterns (3), (5), (6), (8), and (12) in training to reach the accuracy of 0.99. (By Intel CPU i5-8265 with 4 cores and RAM of 32GB). The same approach took the shorter duration of 0.999, 1.004, 1.032, 0.892, and 0.915 s for the SVM model, which also required 33.8 s for training with pattern (1) of all 63 features. The number of features is relative to computational complexity in the modeling for multiclass recognition. In the case of large-amount features, the training duration for iteration in the neural network is much longer than that for classification by dimension separation [48]. The suitable feature sets can be explored for the rehab exercise involving complicated motions, while both complexity and accuracy in computation can be referred to evaluate the model’s capability in recognition.
(5) *The modal decomposition can imply innovative features for recognizing the various motions*. The EMD assisted in extracting the frequency-domain features, which could comprise local characteristics for the allowable ROM due to various subjects, for the modeling [49]. The model enabled the clinical practice to explore the same exercise pattern to recognize different people’s movements. Compared with the modern study of deep learning (DL), which filters the local features from the diverse gaits through the progressive NN-based layers [50], the conventional ML process was also available to achieve the approved recognition efficacy. The data pre-processing was necessary for involving the possible futures in the segment prior to efficiently establishing the recognition model. In addition, the instantaneous energy and frequency, as well as the IMF’s envelopes due to HHT, were suggestive as the training segments for the ML/DL modeling in the future study.

### 4.2. Limitation

The rehabilitation exercises mostly comprise stable and slowly changing movements. The present design for the lower-limb rehabilitation limited the simple and stable exercises on the same plane. The angle-related features were enough for the modeling to reach good recognition. If the physical therapy assigned strenuous exercises for the subject, then the variables derived from acceleration and angular velocity were available for the diverse feature patterns to discover the model.

This study was limited to the budget for purchasing enough wearable sensors before recruiting the participants for the clinical test. Therefore, the development, therefore, focused on the modeling phase to discover the diverse feature patterns for recognizing the exemplified exercises. In advance, the PRR system established by our previous study was improved to track the multiple rehabilitation exercises with AIoT functionalities for the UHM.

The modeling was unable to detect the arbitrary exercise in freestyles because each motion cycle must follow the therapy with the designed schedule. However, the periodical exercise with diverse ROMs of limbs was trainable in the proposed modeling. The system prototype requested the scheduled motion guide that defines the timestamps to capture the exercise’s cyclic segments from the uploaded data for data extraction and feature transformation. If the therapist needs to track the various exercises in a record, then the guide should contain a specified duration (e.g., 30 s) for changing the exercise. In practice, the same rehab exercise was suggestive in each uploaded dataset for effective management.

The proposed system requested either a self-defined exercise schedule or the clinical motion guide to determine the segment’s timestamps at the current phase. In the next phase, the functionality with dynamic time warping will be implemented in the system to detect the periodical signals efficiently. By comparing the literatures for the lower-limb rehabilitation, the program mostly applied the robotic solution that embedded the sensors with a recognition control function, which is similar to wearing the aided device at the fixed position [51,52]. We plan to cooperate with our hospital to implement the model with the human test protocol in the future.

## 5. Conclusions

This study enhanced the PRR system established by our previous study [5,6] with the multiclass recognition models for measuring the hybrid lower-limb rehabilitation motions. The AI-based modeling process comprised the data preprocessing, featuring, machine learning, and validation modules for recognizing the designed rehab exercises. The ML models employed the SVM and ANFIS methods to evaluate the 63 features in the time and frequency domains to generate the diverse recognizable patterns, in which each analytical segment labeled by an exercise included a cycle of the data points. The HHT process was applied to derive the segment’s frequency-domain features involving frequencies and energies in the MHS with respect to the IMFs. As a result, the feature patterns combining the time- and frequency- domain features, such as relative motion angles in y and z axes, and frequency and energy in IMF’s MHS, achieved successful recognition. The suggestive recognition patterns were installed in the prototype of the PRR system, which integrated the AIoT-enabled functions with the MRS, to enable the well-trained ML models for intelligent computation. The AIoT-PRR system with the proposed modeling can be applied to serve the UHM for remotely tracking the rehabilitation of stroke, hemiplegia, and other conditions in the future.

## Figures and Tables

**Table 1:** The lower-limb rehabilitation exercises and the rehabilitated parts [35,36,37].

Ex. No. (Muscle No.)	Start Motion	End Motion
Ex. 1 (1, 2)	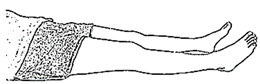	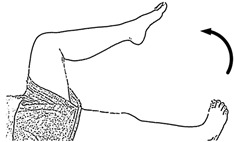
Ex. 2 (3)	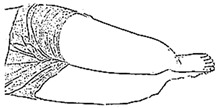	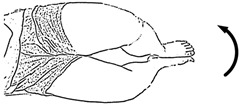
Ex. 3 (4)	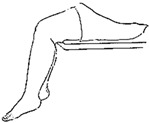	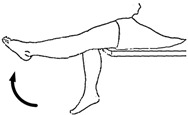
Ex. 4 (5, 6, 7)	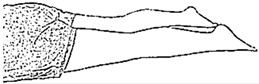	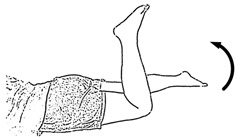
Ex. 5 (8)	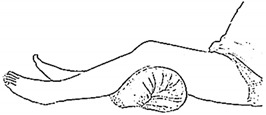	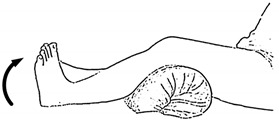
Ex. 6 (9, 10, 11, 12, 13, 14, 15)	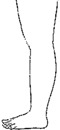	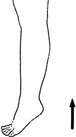

Definition of muscle no. referred to the figures in the “anatomy and physiology” online tutor: 1. Iliacus, 2. Psoas major, as shown in Figure 11.16 (b) from reference [38], 3. Gluteus Medius (cut), 4. Quadratus femoris, 5. Biceps femoris, 6. Semimembranosus, 7. Semitendinosus, as shown in Figure 11.29 from reference [39], 8. Tibialis anterior, 9. Gastrocnemius (lateral head), 10. Gastrocnemius (medial head), 11. Plantaris, 12. Soleus, 13. Tibialis posterior, 14. Flexor digitorum longus, 15. Flexor hallucis longus, as shown in Figure 11.32 from reference [39]. The motion sketches were based on the pictures of the author’s postures.

**Table 2:** The motion guide and schedule designed for the lower-limb exercises.

Ex. No. ^1^	Decomposed Motions and Schedule	Seconds
Ex. 1	(1) Lie flat on the bed	2
	(2) Bend the knee and lift the affected foot to the highest point	2
	(3) Keep the foot raised for a while	5
	(4) Put the foot down and return to the original state	2
Ex. 2	(1) Lie on the side on the bed, place the affected foot on top, bring the feet together, and place the hand on the side of the hip	4
	(2) Open the knees slightly, as long as the hands feel the contraction of the hip muscles	1
	(3) Keep the knees open for a while	5
	(4) Bring the knees together and return to the original state	1
Ex. 3	(1) Sit on the edge of the bed, keep the feet off the ground, keep the waist straight, and do not grasp the edge to exert force	2
	(2) Kick the affected side’s foot straight	2
	(3) Keep the kick straight and stay for a while	5
	(4) Put your feet down Back to the original state	2
Ex. 4	(1) Lie on the bed with a pillow under the chest to support the upper body and keep it elevated	2
	(2) Bend the knee of the affected foot and lift the calf, and the pelvis should not leave the bed	2
	(3) Stay hooked for a period of time	5
	(4) Put the feet down and return to the original state	2
Ex. 5	(1) Sit on the bed, put the hands back on the bed, and place a pillow under the knee joint to support	4
	(2) Tilt up the sole of the affected foot	1
	(3) Keep the bottom of the foot lifted for a period of time	5
	(4) Relax the soles of the feet and return to the original state	1
Ex. 6	(1) Stand by the chair, hold the back of the chair and straighten the torso, open the feet to shoulder-width apart	4
	(2) Stand on tiptoes, do not lean forward torso, and do not turn inward heels	1
	(3) Keep the toe on for a while	5
	(4) Put the heel down and return to the original state	1

^1^ Exercise note: Ex. 1. iliopsoas muscle, Ex. 2. gluteus medius, Ex. 3. quadriceps femoris, Ex. 4. hamstrings, Ex. 5. tibialis anterior, and Ex. 6. triceps surae exercises.

**Table 3:** The feature patterns including the possible variables in time and frequency domains.

No.	Feature Symbol ^1^	Description with the Equation of x-axis Component
1	xsum	Summation of segment data (∑x)
2	xabs_sum	Summation of absolute values of segment data (∑abs(x))
3	xsqr_sum	Summation of square values of segment data ($\sum x^{2}$)
4	xavg	Average of segment data ($\mu =\frac{1}{n}\sum x$)
5	xabs_avg	Average of absolute values of segment data ($\frac{\sum abs\left(x\right)}{n}$)
6	xmedian	Median value of segment data ($\left\{x_{i}\right\},i=\frac{n}{2}$)
7	xquart	Quartile distance of segment data ($\left\{x_{i}\right\},i=\frac{n}{4},\;\frac{3n}{4}\;$)
8	xmin	Minimum of segment data (min(x))
9	xmax	Maximum of segment data (max(x))
10	xrange	max(x)−min(x)
11	xabs_dev_avg	Mean absolute deviation ($\frac{1}{n}\sum abs\left(x-\mu \right))$
12	xstd_dev	The standard deviation of segment data ($\sigma =\sqrt{\frac{1}{n}\sum {\left(x-\mu \right)}^{2}}$)
13	xvar	Variance of segment data ($\;\frac{1}{n}\sum {\left(x-\mu \right)}^{2}$)
14	xskrew	The skewness of segment data distribution (SK = $\frac{\sum {\left(x-µ\right)}^{3}}{\sigma^{3}}$)
15	xkurt	Kurtosis of segment data distribution (KT = $\frac{\sum {\left(x-µ\right)}^{4}}{\sigma^{4}}$)
16	xIMF1_f	The frequency corresponding to the MHS-area centroid of IMF1
17	xIMF2_f	The frequency corresponding to the MHS-area centroid of IMF2
18	xIMF3_f	The frequency corresponding to the MHS-area centroid of IMF3
19	xIMF1_e	The energy corresponding to the MHS-area centroid of IMF1
20	xIMF2_e	The energy corresponding to the MHS-area centroid of IMF2
21	xIMF3_e	The energy corresponding to the MHS-area centroid of IMF3

^1^ Note: each feature symbol includes three-axis components (i.e., the x can be replaced by y and z).

**Table 4:** The critical parameters used in the SVM and ANFIS models.

Parameter	Value
*SVM with polynomial kernel function* ^1^	
Kernel scale parameter	1
Regulation parameter	1
Polynomial order	3
Coding design mode	one-versus-one
Outlier fraction	0~0.001
*ANFIS with subractive clustering method* ^2^	
Cluster influence range	0.8
Squash factor	1.25
Accept ratio	0.5
Reject ratio	0.15
Membership function	Gauss MF

^1^ Solver: Sequential minimal optimization (SMO) or iterative single data algorithm (ISDA). ^2^ Defuzzification: weight average method.

**Table 5:** Definition of the feature patterns and accuracies of the trained models with five-fold cross-validation.

Pattern ID	Feature Set	FEATURE SYMBOL	ML Model ^1^
(1)	All features	21 time- and frequency-domain features for x, y, z axes	SVM, ANFIS (0.99, N/A)
(2)	iIMFj_f, iIMFj_e, (*i* = x, y, z; *j* = 1, 2, 3)	Frequency and energy of IMFs for x, y, z components	SVM, ANFIS (0.75, 0.28)
(3)	y1, z1	sum of y, z components	SVM, ANFIS (0.99, 0.99)
(4)	y2, z2	abs_sum of y, z components	SVM, ANFIS (0.87, 0.71)
(5)	y1, z1, y2, z2	sum and abs_sum of y, z components	SVM, ANFIS (0.99, 0.99)
(6)	y4, z4	avg of y, z components	SVM, ANFIS (0.99, 0.99)
(7)	y5, z5	abs_avg of y, z components	SVM, ANFIS (0.87, 0.79)
(8)	y4, y5, z4, z5	avg and abs_avg of y, z components	SVM, ANFIS (0.99, 0.99)
(9)	iIMF1_f, iIMF1_e, (*i* = x, y, z)	frequency and energy of IMF1 for x, y, z components	SVM, ANFIS (0.58, 0.33)
(10)	iIMF2_f, iIMF2_e, (*i* = x, y, z)	frequency and energy of IMF2 for x, y, z components	SVM, ANFIS (0.58, 0.36)
(11)	iIMF3_f, iIMF3_e, (*i* = x, y, z)	frequency and energy of IMF3 for x, y, z components	SVM, ANFIS (0.61, 0.32)
(12)	y4, z4, y5, z5, iIMF3_f, iIMF3_e, (*i* = y, z)	avg, abs_avg of y, z components; frequency and energy of IMF3 for y, z components	SVM, ANFIS (0.99, 0.99)
(13)	All 21 features of x-axis components	21 time- and frequency-domain features for the x-axis	SVM, ANFIS (0.67, 0.26)
(14)	All 21 features of y-axis components	21 time- and frequency-domain features for the y-axis	SVM, ANFIS (0.94, 0.37)
(15)	All 21 features of z-axis components	21 time- and frequency-domain features for the z-axis	SVM, ANFIS (0.84, N/A)

^1^ The values in the bracket under the SVM and ANFIS represent the average accuracies corresponding to the models.

## Appendix A

**Table A1:** Recognition AUCs of the proposed patterns and models for the subjects’ six exercises.

	Model	Subject A						Subject B					
Pattern	Ex. No.	Ex. 1	Ex. 2	Ex. 3	Ex. 4	Ex. 5	Ex. 6	Ex. 1	Ex. 2	Ex. 3	Ex. 4	Ex. 5	Ex. 6
(1)	SVM_AUC_	1	1 ^1^	1 ^1^	1	1	1	1	1	0.98	0.97	0.97	0.97
	ANFIS_AUC_	-	-	-	-	-	-	-	-	-	-	-	-
(2)	SVM_AUC_	0.95	0.96	0.93	1	0.84	0.89	0.94	0.96	0.85	0.87	0.82	0.64
	ANFIS_AUC_	0.71	0.48	0.51	0.67	0.69	0.41	0.66	0.5	0.49	0.65	0.55	0.55
(3)	SVM_AUC_	1	1	1	1	1	1	1	1	0.91	0.8	0.97	1
	ANFIS_AUC_	1	1	1	1	1	1	1	1	0.95	1	0.96	1
(4)	SVM_AUC_	0.88	0.77	1	1	0.98	1	0.95	0.97	0.75	1	0.77	0.95
	ANFIS_AUC_	0.77	0.72	0.86	0.98	0.74	0.7	0.57	0.89	0.51	0.86	0.57	0.57
(5)	SVM_AUC_	1	1	1	1	1	1	0.98	1	0.92	0.83	1	1
	ANFIS_AUC_	1	1	1	1	1	1	0.99	0.99	0.94	0.87	0.52	0.99
(6)	SVM_AUC_	1	1	1	1	1	1	1	1	0.9	0.81	0.97	1
	ANFIS_AUC_	1	1	1	1	1	1	1	1	0.96	0.98	0.9	1
(7)	SVM_AUC_	0.98	0.93	1	1	0.98	1	0.95	0.97	0.75	1	0.77	0.95
	ANFIS_AUC_	0.9	0.93	0.68	0.98	0.96	0.99	0.84	0.96	0.28	0.91	0.4	0.74
(8)	SVM_AUC_	1	1	1	1	1	1	0.98	1	0.92	0.88	1	1
	ANFIS_AUC_	1	1	1	1	1	1	1	1	0.98	0.83	0.91	1
(9)	SVM_AUC_	0.84	0.74	0.76	0.81	0.66	0.81	0.82	0.89	0.73	0.68	0.6	0.68
	ANFIS_AUC_	0.55	0.6	0.59	0.58	0.54	0.55	0.44	0.64	0.55	0.66	0.53	0.52
(10)	SVM_AUC_	0.89	0.91	0.81	0.99	0.86	0.78	0.85	0.88	0.82	0.93	0.72	0.53
	ANFIS_AUC_	0.53	0.71	0.62	0.73	0.53	0.46	0.59	0.78	0.45	0.68	0.57	0.52
(11)	SVM_AUC_	0.9	0.97	0.87	0.9	0.85	0.83	0.84	0.9	0.88	0.96	0.7	0.61
	ANFIS_AUC_	0.51	0.69	0.7	0.48	0.56	0.53	0.47	0.64	0.71	0.68	0.58	0.48
(12)	SVM_AUC_	1	1	1	1	1	1	1	1	1	1	1	1
	ANFIS_AUC_	1	1	1	1	1	1	1	1	0.96	0.91	0.83	1
(13)	SVM_AUC_	0.76	0.48	0.82	0.87	0.56	0.87	0.93	0.63	0.79	0.71	0.4	0.38
	ANFIS_AUC_	0.53	0.65	0.41	0.59	0.49	0.5	0.67	0.46	0.52	0.43	0.59	0.45
(14)	SVM_AUC_	1	1	0.94	1	0.94	1	1	1	0.91	0.85	0.92	1
	ANFIS_AUC_	0.87	0.94	0.65	0.72	0.49	0.55	0.78	0.94	0.6	0.57	0.68	0.7
(15)	SVM_AUC_	0.99	0.66	0.95	1	0.96	0.97	0.98	0.97	1	1	0.89	0.97
	ANFIS_AUC_	0.84	0.9	0.98	0.93	0.89	0.93	0.92	0.95	0.97	0.76	0.59	0.81

^1^ The initial AUC values regarding Subject A’s Ex.2 and 3 were 0.5 and 0.9, respectively, because of the incorrect or unstable motion postures.
